# Assessment of Radiation-Induced Bladder and Bowel Cancer Risks after Conventionally and Hypo-Fractionated Radiotherapy for the Preoperative Management of Rectal Carcinoma

**DOI:** 10.3390/jpm12091442

**Published:** 2022-08-31

**Authors:** Ekaterini Matsali, Eleftherios P. Pappas, Efrossyni Lyraraki, Georgia Lymperopoulou, Michalis Mazonakis, Pantelis Karaiskos

**Affiliations:** 1 Medical Physics Laboratory, Medical School, National and Kapodistrian University of Athens, 11527 Athens, Greece; 2 Department of Radiotherapy and Oncology, University Hospital of Iraklion, 71110 Iraklion, Greece; 3 1st Department of Radiology, Medical School, National and Kapodistrian University of Athens, 11528 Athens, Greece; 4 Department of Medical Physics, Faculty of Medicine, University of Crete, 71003 Iraklion, Greece

**Keywords:** rectal cancer, radiotherapy, IMRT, VMAT, hypo-fractionation, lifetime attributable risk, second cancer, bladder, bowel

## Abstract

Preoperative management of rectal carcinoma can be performed by employing either conventionally or hypo-fractionated Radiotherapy (CFRT or HFRT, respectively), delivered by Intensity Modulated Radiotherapy (IMRT) or Volumetric Modulated Arc Therapy (VMAT) plans, employing 6 MV or 10 MV photon beams. This study aims to dosimetrically and radiobiologically compare all available approaches, with emphasis on the risk of radiation-induced second cancer to the bladder and bowel. Computed Tomography (CT) scans and relevant radiotherapy contours from 16 patients were anonymized and analyzed retrospectively. For each case, CFRT of 25 × 2 Gy and HFRT of 5 × 5 Gy were both considered. IMRT and VMAT plans using 6 MV and 10 MV photons were prepared. Plan optimization was performed, considering all clinically used plan quality indices and dose–volume constraints for the critical organs. Resulting dose distributions were analyzed and compared. Moreover, the Lifetime Attributable Risk (LAR) for developing radiation-induced bladder and bowel malignancies were assessed using a non-linear mechanistic model, assuming patient ages at treatment of 45, 50, 55 and 60 years. All 128 plans created were clinically acceptable. Risk of second bladder cancer reached 0.26% for HFRT (5 × 5 Gy) and 0.19% for CFRT (25 × 2 Gy) at the age of 45. Systematically higher risks were calculated for HFRT (5 × 5 Gy) as compared to CFRT (25 × 2 Gy), with 6 MV photons resulting in slightly increased LAR, as well. Similar or equal bowel cancer risks were calculated for all techniques and patient ages investigated (range 0.05–0.14%). This work contributes towards radiotherapy treatment protocol selection criteria for the preoperative irradiation of rectal carcinoma. However, more studies are needed to establish the associated radiation-induced risk of each RT protocol.

## 1. Introduction

According to global cancer statistics [1], rectal cancer has been the third most commonly diagnosed cancer (10.0%) and the second most frequent cause of cancer deaths (9.4%) for both sexes. The cumulative risk of dying due to this type of cancer at an age range of 0–74 years is 0.65% and 0.45% for men and women, respectively [2]. Moreover, 41% among new rectal cancer patients reported in 2020 in the USA were aged between 50 and 64 years and 15% were <50 years old [3]. One of the most important predictors of rectal cancer survival is the disease stage at the time of diagnosis [4]. The later the stage of diagnosis, the lower the patient survival [2]. However, because of the early appearance of rectal cancer symptoms, it can be diagnosed at a localized stage [4].

The management of patients with rectal carcinoma is directly related to the rapid development of radiation technology. Radiotherapy (RT) with photon beams combined with surgery is the standard method recommended for tumor control [5]. Especially, preoperative RT is an approach with significant local control, reducing local recurrence and improving survival [5,6,7]. Preoperative RT for rectal cancer control has been applied to patients as young as 26 years old [8]. Regarding the prescribed therapeutic radiation dose, two perspectives exist for selecting the optimal dose fractionation scheme; (i) the conventionally fractionated RT (CFRT) scheme of 50 Gy delivered to the target in 25 daily fractions combined with chemotherapy and (ii) the hypo-fractionated RT (HFRT) approach of 25 Gy delivered in 5 daily fractions. Both schemes have been widely used for the preoperative management of rectal carcinoma [9,10,11]. Hereinafter, the former scheme will be referred to as “CFRT (25 × 2 Gy)” and the latter as “HFRT (5 × 5 Gy)”. It is noted that these fractionation schemes are also mentioned in the literature as Long Course RT (LCRT) and Short Course RT (SCRT), respectively [9,10,11].

Recent technological advances allowed for the clinical introduction of modern dose delivery RT techniques, such as the Intensity Modulated Radiation Therapy (IMRT) and Volumetric Modulated Arc Therapy (VMAT). The former employs radiation fields with static gantry angle but varying intensity. In the latter technique, radiation is delivered in arcs, i.e., the gantry rotates around the patient while delivering beams of varying intensity. Typically, the photon radiation beams used in both techniques for treatments in the pelvic area are 6 MV or 10 MV [12,13,14]. As compared to the conventional 3D conformal RT, both techniques can deliver dose distributions which are more conformal to the target shape and with increased spatial dose gradients outside the target volume. Thus, radiation-induced toxicity (deterministic side effects) to the surrounding critical organs is minimized [15,16,17].

However, RT may also induce stochastic side effects which mainly include development of second cancer to adjacent normal tissue/organs [18,19]. From a radiobiological point of view, radiation-induced second cancer is a multifactor process taking place into the cells after RT. Organs near the target receive inhomogeneous dose distributions and dose–volume effects take place, which are meaningful for the cell mutagenesis. The effect and its probability are associated with the age of the patient at treatment, type of the tissue irradiated, treatment site and volume of the target, the treatment technique employed, beam quality used, as well as other cellular effects (such as the bystander effects and chronic proliferative processes) [19,20]. Dose rate and delivery time have also been reported to affect the radiobiological response of cells [21].

Recent studies have estimated the risk of radiation-induced second cancer after RT for various treatment sites, indicatively the pelvis, breast, lungs and mediastinum [22,23,24,25,26,27,28]. Given that the risk is highly associated with the dose distribution characteristics (dose homogeneity, conformality and gradient), fractionation scheme and anatomy [19,20], this work presents risk estimates specifically for a variety of preoperative rectal carcinoma treatment protocols, commonly employed in clinical practice. In particular, using a non-linear mechanistic model [29] and relevant radiobiological parameters, the risks for developing radiation-induced second cancer in the bladder and bowel are estimated for RT treatments of different (i) delivery techniques (IMRT and VMAT), (ii) photon beam energies (6 MV and 10 MV) and (iii) fractionation schemes [CFRT (25 × 2 Gy) and HFRT (5 × 5 Gy)], assuming that (iv) the patient age at treatment is 45, 50, 55 and 60 years. Moreover, the present study also serves as a dosimetric comparison between all aforementioned treatment approaches.

## 2. Materials and Methods

### 2.1. Patient Cohort

In this study, datasets from sixteen (16) patients (10 males and 6 females) with cancer to the low or intermediate third of the rectum, who underwent preoperative RT, were analyzed retrospectively. Effort was made to involve the youngest patients found in our database (median age at treatment: 55 years).

For each selected case, all relevant Computed Tomography (CT) images, acquired at 120 kVp for RT treatment planning purposes using a Revolution HD scanner (GE Healthcare, Waukesha, WI, USA), were anonymized and exported in Digital Imaging and Communications in Medicine (DICOM) files format. Moreover, the contoured structures created for RT treatment planning were also anonymized and exported in DICOM-RT file format. The corresponding original dose distributions and treatment plans were not exported, as new plans were created retrospectively to ensure consistency in treatment plan optimization and dose constraints requirements.

All datasets were imported in Monaco version 5.10 (ELEKTA, Crawley, UK) treatment planning system (TPS) for treatment planning and dose calculations. Contouring of the Planning Target Volumes (PTVs) was carried out by a radiation oncologist with experience in pelvic tumors, following the guidelines of the STAR-TREC group [30]. Median PTV among the 16 cases was 1014.72 cc. Regarding organs-at-risk (OARs), the bladder, the bowel, and the left and right femoral heads were considered for the purposes of the present study. Median volumes were 180.72 cc, 1359.35 cc, 147.23 cc and 150 cc, respectively. Regarding the bowel, it was contoured according to the recommendations of the Radiation Therapy Oncology Group [31], using the bowel bag technique [31,32], to account for the small bowel loops motion [33]. The rectum, as being the Gross Tumor Volume (GTV), was excluded from the “bowel” structure. In addition to those included in the original plans, all created or modified contours were reviewed and verified by one more expert.

### 2.2. Treatment Protocols, Techniques and Plan Optimization

Both the CFRT (25 × 2 Gy) and the HFRT (5 × 5 Gy) schemes were considered for each case. As mentioned above, for the conventionally fractionated protocol, the prescription dose was 2 Gy per fraction, 5 fractions per week for a total of 25 fractions. For the short scheme (i.e., the HFRT (5 × 5 Gy)), 25 Gy delivered in 5 daily fractions were prescribed [6,17]. For both RT protocols, the prescription dose covered 95% of the PTV. Regarding OAR sparing, Table 1 lists the (clinically used [34,35]) dose constraints considered in this study and strictly met during treatment plan optimization, for each RT fractionation scheme. $V_{n}$ represents the absolute or relative organ volume (cc or %, respectively) which receives a dose of at least n Gy.

Treatment planning and optimization were also performed in Monaco v.5.1 which includes beam models of an Agility linac head (ELEKTA, Crawley, UK) with a 5-mm Multi Leaf Collimator (MLC). 6 MV and 10 MV flattened photon beams have been commissioned, while both IMRT and VMAT treatment delivery techniques are available. 

For the purposes of the present study, IMRT treatment planning involved nine fields with dynamic MLC and gantry angles of 180°, 220°, 260°, 300°, 340°, 20°, 60°, 100°,140°. Regarding the VMAT plans, a double 360° arc technique was used. The first one was defined in the clockwise direction and the second arc set-up was in the counter clockwise. The number of control points was 180 per arc. By defining the appropriate cost functions based on the dose constraints listed in Table 1, asymmetric and different intensity fields/arcs emerged to provide the optimal fit to the shape of the target volume. A dose grid calculation resolution of 3 mm and a minimum segment width of 5 cm were used for treatment planning with both delivery techniques. All relevant dose calculations were performed using the XVMC Monte Carlo dose engine, incorporated in Monaco TPS, with a statistical uncertainty of <1%. 

For each patient, treatment plans with all possible combinations of fractionation scheme, delivery technique and photon energy were prepared, a total of 8 combinations. Specifically, two IMRT and two VMAT plans were initially generated for each patient, using the 6 and 10 MV photon beams on the basis of the CFRT (25 × 2 Gy) protocol (64 plans in total). Two additional plans for each delivery technique using the same photon energies were created implementing the HFRT (5 × 5 Gy) protocol (another 64 plans). A total of 64 IMRT and 64 VMAT plans were produced. Typical Monitor Units (MUs) for the approved CFRT (25 × 2 Gy) IMRT and VMAT plans were 470 and 440 MUs/fraction, respectively. Corresponding MUs for HFRT (5 × 5 Gy) were 1050 and 920 MUs/fraction.

All calculated dose distributions, contoured structures, relevant dose–volume metrics and plan quality indices were exported from the TPS and imported to MATLAB (The MathWorks Inc., Natick, MA, USA) for further analysis, comparison and figure creation. 

Wherever necessary for the purposes described in Section 2.3 and 2.4, comparison using statistical methods was performed in SPSS Statistics v.26 software (IBM, Chicago, IL, USA). Data normality was assessed using the Kolmogorov-Smirnov test. The Paired Samples *T*-test and the Wilcoxon signed-rank test were used for parametric and non-parametric data analyses, respectively. Differences were considered statistically significant at the 95% confidence level, i.e., for a *p*-value of < 0.05. 

### 2.3. Dosimetric Analysis and Comparison

Dosimetric evaluation of all created plans was performed using the corresponding dose–volume histograms (DVHs), derived from the 3D dose distributions calculated by the TPS. All dose–volume metrics listed in Table 1 were recorded for each plan. Moreover, clinically used plan quality indices related to target dosimetry, such as the Heterogeneity Index (*HI*) and Conformity Index (*CI*) were also determined. Specifically, *HI* is expressed by:

(1) $$HI=\frac{D_{5\%}}{D_{95\%}}$$

where $D_{5\%}$ corresponds to the dose that covers the hottest 5% of the PTV and $D_{95\%}$ is the dose that covers 95% of the PTV (i.e., the prescription isodose in this case). The *CI* is calculated by [36]:

(2) $$CI=\frac{TV_{PD}{}^{2}}{TV\cdot VR_{PD}}$$

where $TV_{PD}$ is the volume of target that receives the prescription dose (*PD*), *TV* is the target volume and $VR_{PD}$ is the total volume of the *PD*.

### 2.4. Radiobiological Analysis

As in clinical practice, parts of the bladder and bowel were included within the PTV and, therefore, received relatively high doses which may increase the risk of developing second cancers [37,38]. To estimate the induced risk, the radiobiological non-linear mechanistic model of Schneider et al. [23,29] was used. The model accounts for the effects associated with dose fractionation, cell proliferation and cell killing and has been repeatedly employed in other risk assessment studies [22,23,24,25,26,27,39]. Organ-specific radiobiological parameters related to these effects were also adopted from the literature [23,29] and are listed in Table 2. Dose inhomogeneity within an organ’s volume is accounted for in terms of the Organ Equivalent Dose (OED) concept. OED calculations were performed for each dose distribution (i.e., patient and plan), using the equation [23,29]:

(3) $$OED=\frac{1}{V_{t}}\sum_{i}V_{Di}\frac{e^{-a\prime_{iD_{i}}}}{a\prime_{i}R}\left[1-2R+R^{2}e^{{a^{\prime }}_{i}D_{i}}-{\left(1-R\right)}^{2}e^{-\frac{{a^{\prime }}_{i}R}{1-R}D_{i}}\right]$$

where $V_{t}$ is the total organ (i.e., bladder or bowel) volume, $V_{Di}$ is the organ volume which receives a $D_{i}$ radiation dose, $\alpha \prime_{i}$ is a parameter related to cell killing and *R* is the cell repopulation factor. The $\alpha \prime_{i}$ and *R* parameters are organ-specific (Table 2). The quantities of $V_{t}$ and $V_{Di}$ were obtained from differential DVHs, derived from each dose distribution. The parameter $\alpha \prime_{i}$ was given by the following formula [23,29]:

(4) $$\alpha \prime_{i}=\alpha +\frac{\beta D_{i}}{n}$$

where α and β are the organ-specific linear quadratic parameters and *n* is the number of dose fractions of the treatment protocol (i.e., *n* = 25 for CFRT and *n* = 5 for HFRT). The *R* and α values for the bladder and bowel were also taken from the literature [23,29] (Table 2) for carcinoma.

For each combination of treatment protocol, delivery technique and beam energy, organ-specific OED calculations were performed for all 16 plans in order to obtain the average OED ($OED_{av}$) within the dataset. A total of 8 $OED_{av}$ were calculated for the bladder and another 8 $OED_{av}$ for the bowel. Subsequently, each one was used to find the associated average Excess Absolute Risk ($EAR_{av}$) according to the following formula [23,29]:

(5) $$EAR_{av}=\beta_{EAR}OED_{av}e^{\left[\gamma_{e}\left(age_{e}-30\right)+\gamma_{\alpha }\ln\left(\frac{age_{a}}{70}\right)\right]}$$

where the parameter $\beta_{EAR}$ is the slope of the dose–response curve for radiation-induced cancer at low doses for the Western population [29]. The age-modifying factors $\gamma_{e}$, $\gamma_{\alpha }$ and the parameter $\beta_{EAR}$ were adopted from the literature [23,29] and are also shown in Table 2. The $age_{e}$ is determined as the patients’ age at the time of treatment. For the purposes of this study, four ages were studied; 45, 50, 55 and 60 years [3,4]. $age_{\alpha }$ is the final attained age of 80 years.

Finally, the risk of developing radiation-induced second cancer in the bladder or the bowel is expressed in terms of the average Lifetime Attributable Risk ($LAR_{av}$) [23,29]:

(6) $$LAR_{av}=\sum_{age_{a,\;min}}^{age_{a,\;max}}EAR_{av}\left(age_{e},\;age_{\alpha }\right)\frac{S\left(age_{\alpha }\right)}{S\left(age_{e}\right)}$$

where $age_{\alpha ,min}$ is the patient’s age at treatment ($age_{e}$) plus a cancer risk free interval of 5 years after the exposure. The ratio $\frac{S\left(age_{\alpha }\right)}{S\left(age_{e}\right)}$ represents the probability of a person to survive from $age_{e}$ to $age_{\alpha }$. This probability was adopted from the United States Life Tables [40].

## 3. Results

### 3.1. Dosimetric Analysis and Comparison

**Figure 1:**
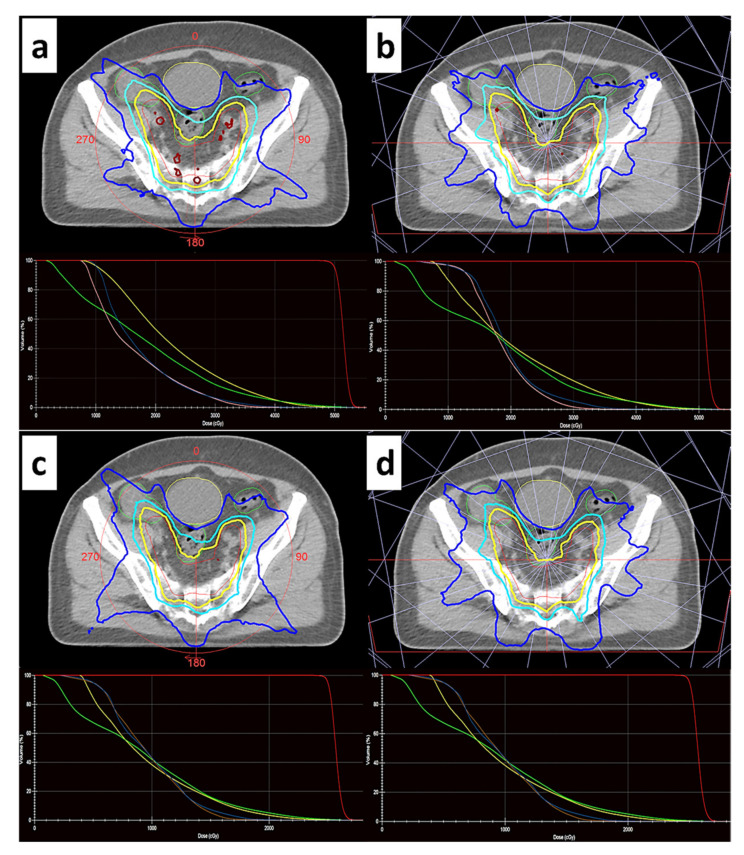
For an indicative patient, an axial slice of the planning CT scan is shown with isodose lines superimposed, for the (**a**,**b**) CFRT (25 × 2 Gy) and (**c**,**d**) HFRT (5 × 5 Gy) fractionation schemes. Corresponding cumulative DVHs are given below. (**a**,**c**) VMAT treatment delivery technique. (**b**,**d**) IMRT treatment delivery technique. The photon beam energy of 10 MV is used for all plans shown. Isoline legend: dark blue: 50%; light blue: 75%; yellow: 95%. DVH and contour legend: red: PTV; yellow: bladder; green: bowel; pink: left femoral head; blue: right femoral head.

The dose distributions calculated by the TPS for an indicative rectal cancer patient subjected to 10 MV IMRT and VMAT therapies are shown in Figure 1. Corresponding cumulative DVHs are also included. A visual inspection of Figure 1 suggests that plan optimization efforts for both planning techniques and fractionation schemes resulted in clinically acceptable dose distributions with sparing of the bladder, bowel and femoral heads, while PTV coverage by the prescription isodose meets the clinical requirements. For a more quantitative analysis of all 128 dose distributions considered, plan quality indices and dose–volume metrics are summarized in Table 3 and Table 4, for the CFRT (25 × 2 Gy) and HFRT (5 × 5 Gy) protocols, respectively. 

Plan quality indices and dose–volume metrics for both PTV and OARs, used for plan evaluation, were similar and clinically acceptable for all generated treatment plans of each treatment protocol (Table 3 and Table 4). 

However, from a statistical point of view, significant differences (i.e., *p* < 0.05) were detected in a few cases. As an instance, CFRT (25 × 2 Gy) pelvic irradiation with 10 MV IMRT led to significantly reduced dose–volume metrics for the bladder and the bowel as compared to the 10 MV VMAT plans. Bladder V_40Gy_ was 13% lower (*p* = 0.001), bowel V_40Gy_ was 10% lower (*p* = 0.02) and bowel V_45Gy_ was 9% lower (*p* = 0.007). Moreover, IMRT delivered with 6 MV photon beams led to a statistically significant reduction in V_35Gy_ value for the bowel as compared to the ones obtained by 10 MV IMRT and VMAT treatments.

Regarding HFRT (5 × 5 Gy), similar conclusions can be drawn. HI and CI values agreed within 1% for all calculated plans, regardless of the delivery technique or photon energy. Again, statistically significant differences were noted in a few cases. For the bladder, statistically significant differences (*p* = 0.003) in V_22Gy_ values were obtained between 6 MV IMRT and 10 MV VMAT. Nevertheless, values deviated by <1%.

**Figure 2:**
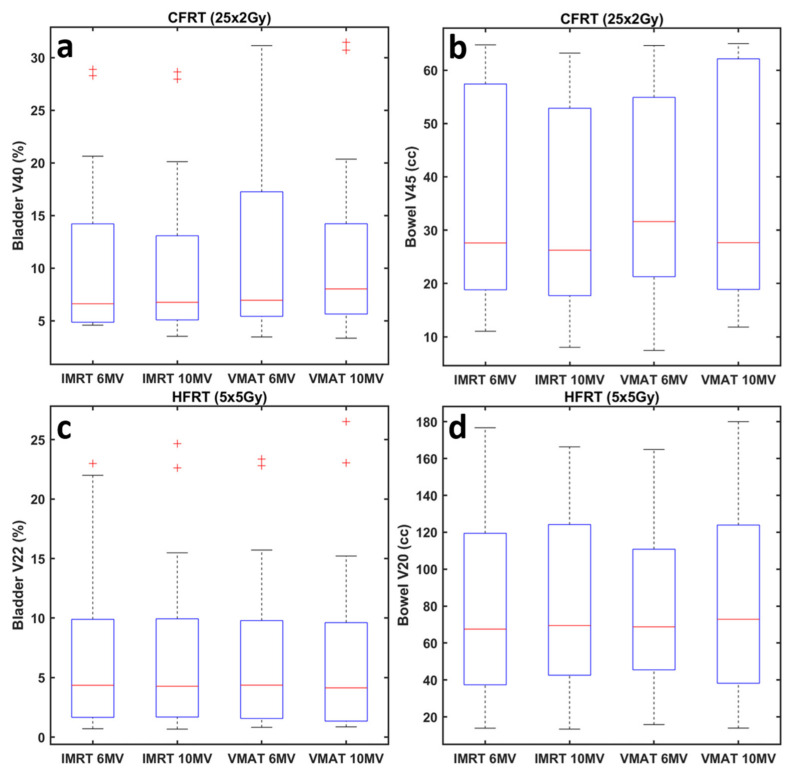
Box-and-whisker plots derived from all dose distributions included in this study, related to clinically used dose–volume metrics for the (**a**,**c**) bladder and (**b**,**d**) bowel. For the (**a**,**b**) CFRT (25 × 2 Gy) treatment protocol, (**a**) V_40_ of the bladder and (**b**) V_45_ of the bowel are shown, while for the (**c**,**d**) HFRT (5 × 5 Gy) (**c**) V_22_ of the bladder and (**d**) V_20_ of the bowel were selected for illustration. Red lines indicate the median value within the corresponding dataset, whereas blue boxes range from the first to the third quartile. Whiskers depict the remaining data or extend up to 1.5 times the interquartile range in either direction. In each dataset, remaining outliers (if any) are shown by the red marks.

All statistically significant differences detected were reviewed and characterized of no clinical importance. To better visualize the spread of the obtained dose–volume metrics, Figure 2 shows box-and-whisker plots derived from all dose distributions and patients. Although a few outliers can be noticed, interquartile ranges and median values for all datasets and metrics are very similar.

### 3.2. Radiobiological Analysis

OED calculations for the bladder and bowel, derived from all available datasets, are summarized in Table 5. Regarding CFRT (25 × 2 Gy), OEDs for bladder and bowel were in the range of 26.0–38.2 cGy and 15.7–16.3 cGy, respectively. Corresponding ranges related to HFRT (5 × 5 Gy) were 40.0–50.7 cGy, and 15.8–17.1 cGy, respectively.

**Figure 3:**
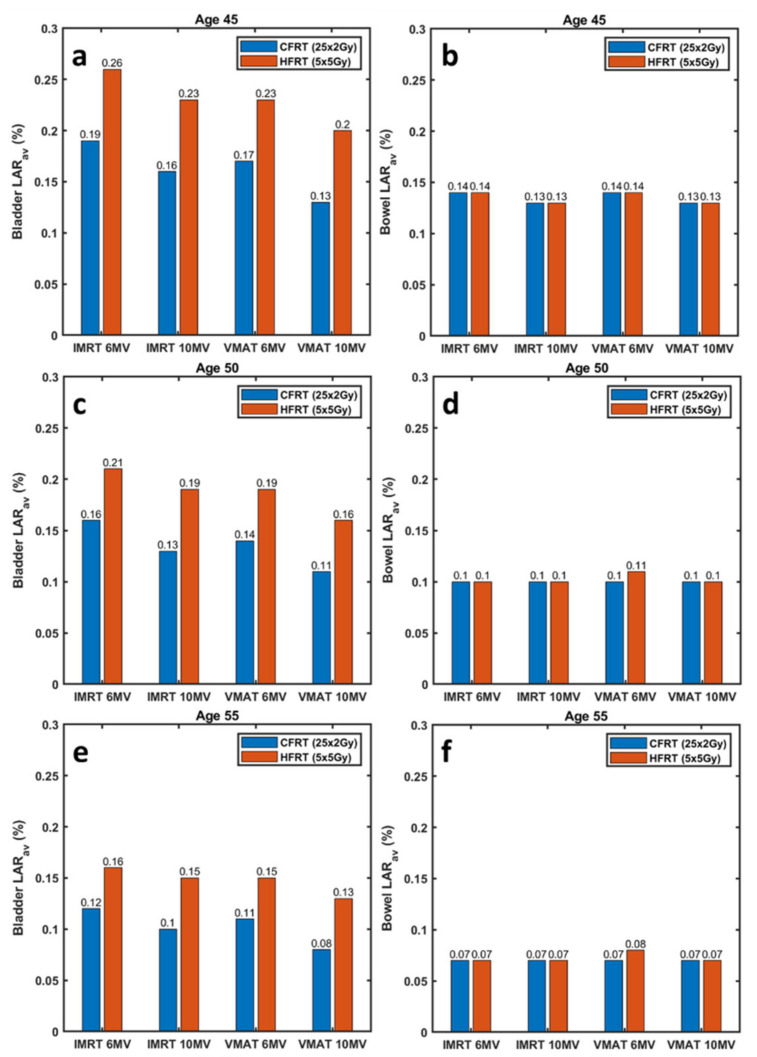

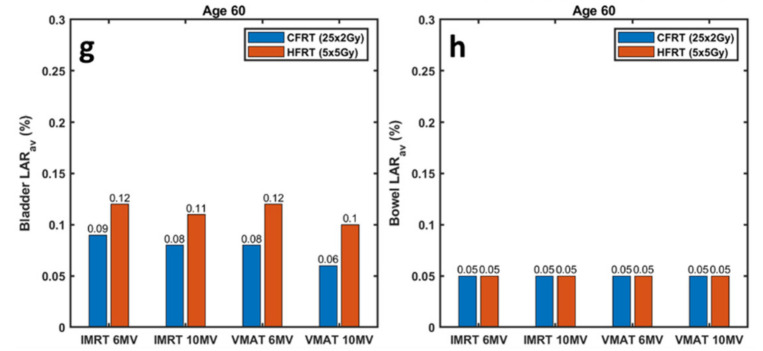
Lifetime Attributable Risk (LAR) estimates for developing radiation-induced second cancer in the (**a**,**c**,**e**,**g**) bladder and the (**b**,**d**,**f**,**h**) bowel, after CFRT (25 × 2 Gy) (blue bars) and HFRT (5 × 5 Gy) (orange bars) treatments at the age of (**a**,**b**) 45, (**c**,**d**) 50, (**e**,**f**) 55 and (**g**,**h**) 60 years.

Using the average OED ($OED_{av}$) for each OAR and dataset (given in Table 5), average LAR ($LAR_{av}$) estimates for the development of radiation-induced second cancer in the bladder and bowel are illustrated in Figure 3. Four ages at treatment were considered; 45, 50, 55 and 60 years old. CFRT (25 × 2 Gy) and HFRT (5 × 5 Gy) are shown side-by-side to assist comparison. For the youngest age considered, LAR for bladder cancer reached 0.26% corresponding to HFRT (5 × 5 Gy) treatment using 6 MV IMRT fields (Figure 3a). Risk of bowel cancer is systematically lower (compared to bladder), not exceeding 0.15% for all ages investigated (Figure 3b). In all datasets, risks related to HFRT (5 × 5 Gy) were higher or at least equal to corresponding risks associated with CFRT (25 × 2 Gy) treatment protocols. This observation is more pronounced for bladder cancer risk and younger ages at treatment. 

## 4. Discussion

Creating treatment plans for two fractionation schemes, two treatment delivery techniques and two photon beam energies allowed for the radiobiological analysis and second cancer risk estimates of a wide variety of treatment approaches/protocols, commonly employed in clinical practice. Thus, this study covers the vast majority of available RT approaches.

In addition to radiobiological assessment, this work also served as a dosimetric comparison study between treatment delivery techniques (i.e., IMRT and VMAT) and photon energies (i.e., 6 MV and 10 MV), for two fractionation schemes. All evaluated dose distributions resulted in clinically acceptable *CI* and *HI* (Table 3 and Table 4). With respect to OAR sparing, dose–volume metrics were similar for all treatment approaches investigated for the same fractionation scheme (Figure 2, Table 3 and Table 4). Although in a few cases statistically significant differences between evaluated datasets were obtained, deviations were very limited and were of no clinical importance. Therefore, for a given fractionation scheme our results suggest that all four RT approaches (i.e., 6 MV IMRT, 10 MV IMRT, 6 MV VMAT and 10 MV VMAT) can be considered acceptable for clinical use [34,35], and of equal effectiveness from a dosimetric point of view. Any attempts to determine the most effective approach were inconclusive. Previous studies have demonstrated the relationship between the dose–volume metrics for the OARs and the severity of side-effects [15,30,41,42]. Based on our results and from DVH analyses, acute or late gastrointestinal toxicity due to bowel exposure, urinary toxicity due to bladder exposure or complications in the femoral heads are not expected using any of the RT approaches investigated. However, it is not uncommon RT treatments that meet the clinical dose constraints still cause toxicity. CFRT (25 × 2 Gy) and HFRT (5 × 5 Gy) have been compared in clinical trials, as well, although different chemotherapy regimens and/or time delay till surgery have been investigated [9,43,44,45,46,47]. In terms of treatment-related side-effects, the Polish I trial reported increased acute toxicity in CFRT (25 × 2 Gy) but late effects did not differ significantly [9]. Similar findings were reported by the Polish II and Tasman group trials [44,45]. However, according to the findings of the Stockholm III trial, acute radiation-induced toxicity is associated with the time delay to surgery [47].

The main goal of this study was to calculate the risk of radiation-induced second cancer in the bladder and bowel for a variety of RT approaches, commonly employed in clinical practice, from a comparative perspective. Based on the results shown in Figure 3, for both treatment delivery techniques (IMRT and VMAT) and irrespectively of the fractionation used [CFRT (25 × 2 Gy) or HFRT (5 × 5 Gy)], the use of 6 MV photons resulted in slightly increased LAR compared to 10 MV plans. CFRT (25 × 5 Gy) and HFRT (5 × 5 Gy) plans resulted in equal cancer risk of the bowel (Figure 3b,d,f,h). 

On the other hand, the bladder cancer risk of both IMRT and VMAT and independently of the energy used was found considerably higher for the HFRT (5 × 5 Gy) scheme compared to that estimated for the conventional fractionation (Figure 3a,c,e,g). Although this remark applies to all four ages investigated, the increased bladder cancer risk with HFRT (5 × 5 Gy), as compared to CFRT (25 × 2 Gy), is more pronounced for the younger ages at treatment. One would expect that the increased total dose in CFRT (25 × 2 Gy) plans would result in higher OED values in the bladder and bowel, as compared to the 25 Gy of HFRT (5 × 5 Gy) plans. However, due to plan optimization efforts to achieve rapid dose falloff within the critical organs, very small parts of the bladder and bowel receive doses close to the prescription dose (Table 3 and Table 4). Thus, the lower isodoses dominate OED and LAR calculations in both fractionation schemes. However, it has been demonstrated that for a theoretical homogeneous irradiation of a normal tissue, increasing the fraction size for the same Biologically Effective Dose (BED) results in reduced second cancer risks [48]. This effect is mitigated in clinical plans because of the spatial modulation of the dose, achieving a rapid dose falloff outside the target and sparing adjacent OARs. In support of the latter remark, Zwahlen et al. [39] also estimated the cancer risk from VMAT treatments for patients with rectal cancer. The authors reported mean bladder cancer risks of 0.2151% and 0.2260% for the long (25 fractions) and short (5 fractions) course RT scheme, respectively (Table 2 in Zwahlen et al. [39]). The corresponding small bowel cancer risks were found to be 0.1328% and 0.1028%, respectively. Although, the authors used the actual patients’ ages at treatment, the values found in that study are in close agreement with present LAR estimations and verify one of our conclusions; HFRT (5 × 5 Gy) treatments are associated with slightly higher bladder cancer risk compared to CFRT (25 × 2 Gy). 

Over the last decades, second cancer incidents have dramatically increased among cancer survivors and RT has been clearly identified as a contributing factor [49]. However, based on rectal cancer cases follow-up, Martling et al. reported only a slight increase in second cancer incidents for patients who received RT compared to those who did not and only for the longer follow-up group (up to 31 years) [18]. In another follow-up study, Kendal et al. also reported an infrequent appearance of second cancers with respect to the background incidence, after RT for rectal cancer [50]. Wiltink et al. concluded that previous RT for rectal cancer did not result in a higher risk of second cancer [51]. Using a cohort of 29027 patients, the study of Rombouts et al. [52] reported that RT for rectal cancer might even have a protective effect against the development of second cancer to the prostate, in accordance with relevant remarks in Martling et al. [18]. On the other hand, gynecological second malignancies occurred more often in women subjected to rectum RT compared to the ones who did not [52]. The rather low LAR values obtained in the present work support the rare or undetectable radiation-induced second cancer incidents after rectal RT by the clinical patient follow-up studies. Moreover, it should be noted that contemporary RT treatment protocols (e.g., VMAT or HFRT (5 × 5 Gy)) are relatively new as compared to the time needed to develop and detect radiation-induced second cancer incidents in clinical trials.

A number of limitations related to this study are noteworthy. First, results relied on a cohort of 16 patients. Although effort was put to increase the number of patients, inclusion of challenging or extreme cases could potentially bias the overall results and limit the applicability of our conclusions. Nevertheless, the number of patients included in our study is typical compared to previously published works retrospectively analyzing plans and reporting on radiation-induced second cancer (range: 5 up to 26 patients) [22,23,24,25,26,27,28,39]. Thus, presented results can be considered indicative, but only for typical preoperative rectal cancer cases, and more studies are needed to verify the estimated risks for each RT protocol/approach. Moreover, all dose distributions analyzed were derived from the TPS and, therefore, potential dosimetric and/or spatial inaccuracies by the treatment delivery unit or the TPS (e.g., sub-optimal beam modeling) were not accounted for. Despite the fact that our treatment protocols are periodically benchmarked by implementing stringent quality control checks, an indicative number of plans have been validated by employing patient-specific quality assurance procedures, according to the clinical workflow. Performing patient-specific dosimetry for all 128 plans created would have been redundant, given the retrospective nature of this study. Furthermore, accuracy of LAR calculations for the bladder and bowel relies on the accuracy of a specific radiobiological model (Schneider et al. [29]) and relevant organ-specific radiobiological parameters (i.e., $\beta_{EAR}$, $\gamma_{\alpha }$, $\gamma_{e}$, α, β and $\frac{\alpha }{\beta }$ in Table 2). Although the employed model is well-established and has been widely used in previous works [22,23,24,25,26,27,28,39], as a comparative study, it can be argued that relative LAR estimates between fractionation schemes and delivery techniques are partly immune to systematic uncertainties of the radiobiological model. The dosimetric evaluation and estimation of second cancer risks were obtained by analyzing plans with 6 MV and 10 MV photon beams. Higher photon energies of 15 or 18 MV were not considered in this study due to the occurrence of secondary neutrons. The presence of neutrons may alter the radiation exposure of the OARs. Kry et al. [53] found that the risk of fatal second malignancy from radiotherapy emitting 15 or 18 MV photons is increased compared to the corresponding one from with 6 and 10 MV photons. Radiobiological assessment of other photon beam qualities or particle RT is beyond the scope of this work. Lastly, the role of chemotherapy, often combined with CFRT (25 × 2 Gy), as a contributing factor for carcinogenesis should also be studied for a comprehensive risk assessment [49].

For a given rectal cancer case, treatment protocol selection criteria are based on the clinical condition of the patient, cancer staging, expected local and distal control of the tumor, any potential radiation-induced toxicity (i.e., deterministic effects), patient co-operation, technological equipment involved, as well as availability, advantages and limitations of the commissioned modalities, treatment planning techniques, photon beam energies and delivery units [54,55]. Other social, geographical and financial issues are also taken into account during selection of the treatment protocol. Overall results of this work suggest that the RT-related risk of developing second cancer to OARs should also be considered. This is more pronounced for younger patients. However, more studies are needed before the risk of radiation-induced second cancer is taken into account as an additional criterium for RT treatment protocol selection.

## 5. Conclusions

This study demonstrates that CFRT (25 × 2 Gy) and HFRT (5 × 5 Gy) employing IMRT and VMAT treatment delivery techniques and 6 MV or 10 MV photon beams resulted in clinically acceptable dose distributions, considering both target dosimetry and OAR sparing. Plan quality indices and dose–volume metrics were found similar among all datasets investigated. Although statistically significant differences were obtained for a few metrics and datasets, differences were very limited and of no clinical importance. Thus, from a dosimetric point of view, determination of the most effective RT approach for preoperative rectal cancer irradiation was inconclusive.

On the other hand, the radiobiological analysis revealed that the risk of radiation-induced second cancer to the bladder increases if a HFRT (5 × 5 Gy) is considered, as compared to CFRT (25 × 2 Gy). The increase is more pronounced in younger ages and LAR reached 0.26% for a patient treated with HFRT (5 × 5 Gy) delivered with 6 MV IMRT beams at the age of 45 years. Corresponding risk of CFRT (25 × 2 Gy) was 0.19%. Another remark based on LAR estimates is that 6 MV photon beams resulted in slightly increased risks for bladder cancer compared to 10 MV beams, for both fractionation schemes and delivery techniques considered. Regarding cancer risk to the bowel, similar or equal LARs were calculated for all datasets analyzed and patient ages investigated.

This work contributes towards RT treatment approach/protocol selection criteria for the preoperative management of rectal cancer. However, more studies are needed to verify the estimated second cancer risks, prior to establishing the relevant criteria.

## Figures and Tables

**Table 1:** Dose constraints considered and strictly met during plan optimization for the organs at risk (OARs), for the CFRT (25 × 2 Gy) and the HFRT (5 × 5 Gy) treatment protocols.

OAR	CFRT (25 × 2 Gy)	HFRT (5 × 5 Gy)
Bowel	$V_{35Gy}$ < 180 cc	$V_{20Gy}$ < 200 cc
	$V_{40Gy}$ < 100 cc	
	$V_{45Gy}$ < 65 cc	
Bladder	$V_{40Gy}$ < 40%	$V_{22Gy}$ < 35%
	$V_{45Gy}$ < 15%	
Femoral Heads	$V_{40Gy}$ < 40%	$V_{15Gy}$ < 40%
	$V_{45Gy}$ < 25%	

Abbreviations: CFRT: Conventionally Fractionated Radiation Therapy; HFRT: Hypo-Fractionated Radiation Therapy.

**Table 2:** Organ-specific radiobiological model parameters used for the calculation of OED and EAR for developing radiation-induced second cancer in the bowel and bladder. The model parameters correspond to carcinoma [23,29].

Radiobiological Parameter	Bladder	Bowel
*R*	0.06	0.09
*α* (Gy^−1^)	0.219	0.591
*α*/*β* (Gy)	3.0	3.0
*β*(1/Gy^2^)	0.073	0.197
$\beta_{EAR}$$(10^{4}\;PY\;GY$)	3.8	10
$\gamma_{e}$	−0.024	−0.056
$\gamma_{\alpha }$	2.38	6.9

Abbreviations: OED: Organ Equivalent Dose; EAR: Excessive Absolute Risk.

**Table 3:** Mean ± 1 standard deviation of the clinically used plan quality indices and dose–volume metrics, related to the CFRT (25 × 2 Gy) treatment protocol for preoperative rectal cancer IMRT and VMAT irradiation with 6 MV or 10 MV photon beams.

Structure	Dosimetric Index	IMRT		VMAT	
		6 MV	10 MV	6 MV	10 MV
PTV	*HI*	1.05 ± 0	1.05 ± 0	1.06 ± 0.01	1.06 ± 0.01
	*CI*	0.84 ± 0.01	0.85 ± 0.01	0.84 ± 0.02	0.84 ± 0.01
Bladder	V_40Gy_ (%)	10.85 ± 8.35	10.52 ± 8.18	11.90 ± 9.07	11.85 ± 8.79
	V_45Gy_ (%)	5.43 ± 4.85	5.23 ± 4.68	5.70 ± 4.41	5.52 ± 4.39
Bowel	V_35Gy_ (cc)	118.10 ± 31.90	129.47 ± 37.25	131.42 ± 38.39	137.42 ± 38.12
	V_40Gy_ (cc)	62.61 ± 18.64	63.61 ± 20.37	68.15 ± 23.56	70.23 ± 22.91
	V_45Gy_ (cc)	33.97 ± 20.26	32.60 ± 19.40	25.45 ± 19.58	35.45 ± 20.41

Abbreviations: PTV: Planning Target Volume; OAR: Organ at Risk; CFRT: Conventionally Fractionated Radiation Therapy; HFRT: Hypo-Fractionated Radiation Therapy; *HI*: Homogeneity Index; *CI*: Conformity Index; IMRT: Intensity Modulated Radiation Therapy; VMAT: Volumetric Modulated Arc Therapy.

**Table 4:** Mean ± 1 standard deviation of the clinically used plan quality indices and dose–volume metrics, related to the HFRT (5 × 5 Gy) treatment protocol for preoperative rectal cancer IMRT and VMAT irradiation with 6 MV or 10 MV photon beams.

Structure	Dosimetric Index	IMRT		VMAT	
		6 MV	10 MV	6 MV	10 MV
PTV	*HI*	1.05 ± 0	1.05 ± 0	1.06 ± 0.01	1.06 ± 0.01
	*CI*	0.84 ± 0.01	0.85 ± 0.01	0.84 ± 0.02	0.84 ± 0.01
Bladder	V_22Gy_ (%)	7.07 ± 2.90	7.10 ± 2.45	7.02 ± 2.87	7.10 ± 2.00
Bowel	V_20Gy_ (cc)	79.50 ± 49.06	81.00 ± 48.73	81.26 ± 46.43	84.27 ± 51.57

Abbreviations: PTV: Planning Target Volume; HFRT: Hypo-Fractionated Radiation Therapy; *HI*: Homogeneity Index; *CI*: Conformity Index; IMRT: Intensity Modulated Radiation Therapy; VMAT: Volumetric Modulated Arc Therapy.

**Table 5:** (Average ± 1 standard deviation) Organ Equivalent Dose ($OED_{av})$ calculations derived from CFRT (25 × 2 Gy) and HFRT (5 × 5 Gy) treatment plans for preoperative rectal cancer irradiation using IMRT and VMAT, and 6 MV or 10 MV photon beams.

		$OED_{av}\;(\mathrm{cGy})$			
		CFRT (25 × 2 Gy)		HFRT (5 × 5 Gy)	
Delivery Technique	Photon Beam Energy	Bladder	Bowel	Bladder	Bowel
IMRT	6 MV	38.2 ± 9.0	16.5 ± 4.0	50.7 ± 16.1	15.8 ± 4.3
	10 MV	31.7 ± 6.7	15.8 ± 4.2	46.2 ± 15.9	15.8 ± 4.3
VMAT	6 MV	33.7 ± 8.1	16.3 ± 4.0	46.5 ± 13.8	17.1 ± 4.1
	10 MV	26.0 ± 5.7	15.7 ± 3.7	40.0 ± 10.6	15.8 ± 4.3

Abbreviations: CFRT: Conventionally fractionated Radiation Therapy; HFRT: Hypo-Fractionated Radiation Therapy; IMRT: Intensity Modulated Radiation Therapy; VMAT: Volumetric Modulated Arc Therapy.

## Data Availability

The data presented in this study are available on request from the corresponding author.
